# Childhood Obesity, Cortical Structure, and Executive Function in Healthy Children

**DOI:** 10.1093/cercor/bhz257

**Published:** 2019-10-24

**Authors:** Lisa Ronan, Aaron Alexander-Bloch, Paul C Fletcher

**Affiliations:** 1 Department of Psychiatry, University of Cambridge, Cambridge CB2 8HA UK; 2 Department of Child and Adolescent Psychiatry and Behavioral Science, Children’s Hospital of Philadelphia, PA 19104, USA; 3 Cambridgeshire and Peterborough NHS Foundation Trust, Cambridge CB21 5EF, UK; 4 The Wellcome-MRC Institute of Metabolic Science-Metabolic Research Laboratories (IMS-MRL), University of Cambridge, Cambridge CB2 0QQ, UK

**Keywords:** ABCD, childhood obesity, cortical thickness, executive function, prefrontal cortex

## Abstract

The development of executive function is linked to maturation of prefrontal cortex (PFC) in childhood. Childhood obesity has been associated with changes in brain structure, particularly in PFC, as well as deficits in executive functions. We aimed to determine whether differences in cortical structure mediate the relationship between executive function and childhood obesity. We analyzed MR-derived measures of cortical thickness for 2700 children between the ages of 9 and 11 years, recruited as part of the NIH Adolescent Brain and Cognitive Development (ABCD) study. We related our findings to measures of executive function and body mass index (BMI). In our analysis, increased BMI was associated with significantly reduced mean cortical thickness, as well as specific bilateral reduced cortical thickness in prefrontal cortical regions. This relationship remained after accounting for age, sex, race, parental education, household income, birth-weight, and in-scanner motion. Increased BMI was also associated with lower executive function. Reduced thickness in the rostral medial and superior frontal cortex, the inferior frontal gyrus, and the lateral orbitofrontal cortex partially accounted for reductions in executive function. These results suggest that childhood obesity is associated with compromised executive function. This relationship may be partly explained by BMI-associated reduced cortical thickness in the PFC.

## Introduction

Although the rise in incidence of childhood obesity appears to have plateaued in some developed nations, the condition is still estimated to effect 124 million children worldwide (NCD-RisC 2017) or an estimated 1 in 3 children in the US (Ogden et al. 2014). Children who are overweight or obese are more likely to become obese adults, and have an increased risk of poorer health outcomes in later life including diabetes, heart disease, cancer, and overall mortality (Biro and Wien 2010).

Like adult obesity (Graham et al. 2014; Yang et al. 2018), childhood obesity has been linked to impairments in executive functioning (Maayan et al. 2011; Reinert et al. 2013; Yau et al. 2014; Ross et al. 2015; Alarcón et al. 2016; Li et al. 2018), although studies produce conflicting results (Gunstad et al. 2008). Executive function is an umbrella term for several different cognitive dimensions, including inhibitory control, decision-making, working memory, and reward sensitivity, broadly referring to a set of processes that enable planning, problem-solving, flexible reasoning, and regulation of behaviors and emotions. Children who are overweight or obese generally score lower on various measures of executive function (Liang et al. 2014), including working memory (Riggs et al. 2012), reward-sensitivity (Verbeken et al. 2012) and inhibitory control (Guerrieri et al. 2008; Verbeken et al. 2009).

At a biological level, various hypotheses exist relating executive functions to body mass index (BMI). One prominent theory is that the role of executive function in planning and decision-making, response inhibition, and reward evaluation influences food intake (Gluck et al. 2017), contributing to increased BMI. Prospective studies of bariatric patients following surgery support this association (Spitznagel et al. 2013), as do neuroimaging investigations. For example, functional magnetic resonance imaging studies have demonstrated that the dorsolateral prefrontal cortex (DPFC) is differentially activated in obesity in both adults (Le et al. 2006) and children (Davids et al. 2010; Reinert et al. 2013). This region is critical to cognitive control over eating (Gluck et al. 2017), as well as reward-motivated behavior via its links to the mesolimbic and mesocortical regions of the brain. Other regions of the prefrontal cortex (PFC) such as the orbitofrontal cortex (OFC) have also been linked to obesity. The OFC is involved in inhibition and reward processing (Gehring and Willoughby 2002) and has been shown to be differentially activated in lean and obese people depending on the level of satiety (Del Parigi 2010), including in children and adolescents (Holsen et al. 2005; Batterink et al. 2010; Bruce et al. 2010; Stice et al. 2010). This region is also implicated in the response to food stimuli (Killgore and Yurgelun-Todd 2005) in both children (Holsen et al. 2005) and adults (Kringelbach 2005). The ventral lateral PFC, linked to impulsivity, has also been implicated in obesity (Batterink et al. 2010). More generally, children with lower levels of executive abilities are more likely to be sedentary and have higher rates of snack consumption (Liang et al. 2014), and are less likely to benefit from weight-loss interventions (Eichen et al. 2018). While these studies support a link between executive function and BMI, the direction of causality is unclear. For example, there is compelling evidence suggesting that the low-grade inflammatory response that characterizes obesity may have a causal impact on the brain and impair executive function (Shields et al. 2017; Yang et al. 2018). Supporting this causal pathway, studies of postsurgical executive function in bariatric patients have demonstrated that weight loss is correlated with an improvement in cognitive abilities (Alosco et al. 2014a).

In summary, while the direction of causal association between BMI and executive function is not well understood, neuroimaging studies support the hypothesis that cortical structure and function are important to characterizing the relationship between executive function and BMI. Various investigations have linked adolescent obesity with changes in grey matter volume, connectivity, and reduced cortical thickness, commonly in prefrontal regions known to be associated with executive function (Maayan et al. 2011; Alosco et al. 2014b; Yau et al. 2014; Gupta et al. 2015; Ross et al. 2015). In addition, high fat diets have been linked to changes in microRNA expression related to axonal guidance in the PFC of adolescents (Labouesse et al. 2018). Thus, there is a demonstrable association in adolescents between BMI and cortical structure in regions associated with executive function. Whether such structural changes mediate the relationship between BMI and executive abilities is not established. Late childhood and early adolescence is a critical period for the emergence and consolidation of executive function, which is strongly linked to maturation of the PFC (Gray et al. 2003; Frangou et al. 2004; Tamnes et al. 2013). This maturation is characterized by reduced cortical thickness (Sowell et al. 2004; Shaw et al. 2006), the consolidation of regional activity (Durston et al. 2006), and the emergence of more comprehensive and extensive network connections (Ezekiel et al. 2013). An important question therefore is whether childhood obesity is characterized by structural changes in cortical regions important for executive function at this critical developmental period and further, whether these structural changes mediate the link between BMI and differences in executive function observed in childhood.

We sought to address this question using data from the NIH Adolescent Brain and Cognitive Development (ABCD) dataset (Jernigan and Brown 2018) of *n* = 2700 children between the ages of 9 and 11 years. Specifically we related measures of cortical thickness to measures of executive function and BMI, and further examined whether cortical thickness confounds the observed relationship between these traits. We chose to focus on measures of cortical thickness as cortical thinning in childhood has been linked to the emergence of executive function (Kharitonova et al. 2013; Bathelt et al. 2018), cortical thickness changes have been linked to childhood obesity (Maayan et al. 2011; Reinert et al. 2013; Alosco et al. 2014b; Yau et al. 2014; Ross et al. 2015; Medic et al. 2016), and previous studies in adulthood have demonstrated that cortical thickness in the PFC mediates the relationship between executive function and BMI (Lavagnino et al. 2016).

## Methods and Materials

### Subjects

A total of 3923 children aged 9–11 years from the ABCD dataset were initially included (10.15154/1504466). The ABCD dataset is a longitudinal study of over 10 000 children recruited from 21 centers throughout the US, with participants largely recruited through the school system. Sampling plans and recruitment procedures based on considerations of age, gender, race, socio-economic status, and urbanicity were designed to reflect the sociodemographics of the US. Details of recruitment and study design are described elsewhere (Garavan et al. 2018). Details of demographic, physical, and mental health assessments are described elsewhere (Barch et al. 2018).

BMI was based on measures of height and weight, which were taken as the average of up to 3 separate measures. BMI was calculated as weight in lbs divided by height in inches squared, multiplied by 703 (Eq. (1)).

(1) \begin{equation*} \mathrm{BMI}=703\times \frac{\mathrm{weight}\left(\mathrm{lbs}\right)}{\mathrm{height}\ {\left(\mathrm{in}\right)}^2} \end{equation*}

BMI *z*-scores (BMI*_z_*) were defined using lookup tables from the Center of Disease Control 2001 (CDC Growth Charts 2018), where BMI was adjusted for sex and age. Subjects with a diagnosis of ADHD (*n* = 536), autism spectrum disorder (*n* = 49), schizophrenia (*n* = 2), intellectual disability (*n* = 2), and diabetes (*n* = 9) were excluded from analysis.

Additional analyses were carried out using measures of waist circumference and waist-to-height ratio in place of BMI.

### Imaging Protocols

Imaging protocols for the ABCD dataset are described elsewhere (Casey et al. 2018), and were harmonized for three 3T scanner platforms (Siemens Prisma, General Electric 750 and Philips) used across the 21 data acquisition sites.

### Cortical Reconstruction and Brain Structural Measures

Cortical reconstructions were carried out using FreeSurfer v5.3.0 (Dale et al. 1999; Fischl et al. 1999a, 1999b), as part of the initial baseline processing of the ABCD dataset. Reconstructions were visually inspected for quality control purposes. Only those reconstructions deemed of sufficient quality were included in this study. Based on these surface reconstructions, cortical thickness (Fischl and Dale 2000) values were processed for the Deskian-Killiany atlas (Desikan et al. 2006), with data unavailable for 3/36 regions per hemisphere (omitted regions included “unknown”, “corpus callosum”, and “insula”). Derived results per individual per region were provided as part of the ABCD curated annual release 1.0 (DOI 10.15154/1412097).

### Executive Function

Participants involved in the ABCD study participated in a battery of tests designed to test their executive function. An overview of the baseline neurocognition battery is described elsewhere (Luciana et al. 2018). Tests were based on the National Institute of Health (NIH) Toolbox (https://nihtoolbox.desk.com). A composite score of executive function was generated based on results of several tests, namely the Flanker inhibitory control and attention test, the dimensional change card sort test, the picture sequence memory test, the list sorting working memory test, and the pattern comparison processing speed test (Akshoomoff et al. 2013, 2014, 2018). The age-corrected standard scores for each test were based on a normative sample of 2917 children and adolescents (Casaletto et al. 2015). The composite score was derived by averaging the standard scores of each of the measures and then deriving standard scores based on this new distribution. These age-corrected composite scores were used in subsequent analysis. Full data were available for *n* = 2352 subjects (*n* = 1802, 352 and 702 for lean, overweight and obese, respectively).

### Statistical Analysis

We conducted a mediation analysis to determine whether cortical thickness mediated the relationship between BMI and executive function. As part of this analysis, we determined the following relationships using multivariate methods: regression of executive function on BMI, regression of regional cortical thickness on BMI, and regression of executive function on cortical thickness. Subsequently, we determined whether regional cortical thickness (mediator) was a significant predictor of executive function (the dependent variable) in a model that also included BMI (the independent variable) (Baron and Kenny 1986). We used Mahalanobis distance to identify and remove outliers in all regression models, and false discovery rate (FDR) methods (Benjamini and Hochberg 1995) were used to correct cortical results for multiple comparisons. Standardized regression coefficients were reported.

Mediation was conducted using the “mediation” package in R (Tingley et al. 2014). We assessed the significance of our mediation models using bootstrapping methods to increase power (Hayes 2009), with 1000 bootstrap samples used to generate 95% confidence intervals for the indirect effect. All analyses were conducted in R (v.3.3.3).

#### Covariates

Puberty is known to influence brain development, and pubertal hormones such as dehydroepiandrosterone (DHEA) have been linked to changes in cortical thickness between the ages of 4 and 13 years (Nguyen et al. 2013). To account for the possible confounding effects of the age of onset of puberty, we included salivary DHEA levels (Uban et al. 2018) as a covariate in our analysis. Birth weight was also included as a nuisance variable, as studies have indicated that this may play a role in intelligence scores at 11 years (Korpela et al. 2018) and has been demonstrated to be significantly predictive of childhood obesity (Biro and Wien 2010; Glavin et al. 2014). We also included estimates of head movement during scanning. Such micromotions have been demonstrated to be genetically correlated with BMI (Hodgson et al. 2017) and associated with biases in MR-derived parameters of cortical structure (Alexander-Bloch et al. 2016). For these reasons, frame-wise displacement (FWD) derived from resting-state data was adopted as an estimate of average head motion and included as a covariate. We also included brain volume and a self-reported measure of physical activity, which was recorded as the number of days in the week prior to interview where the subject had been moderately physically active for more than 60 min. Finally, we included covariates of household income, race, and parental education in our analysis as these have been demonstrated to be associated with BMI (Strauss and Knight 1999).

## Results

Our results support statistically significant associations between BMI and executive function, between BMI and cortical thickness, and between cortical thickness and executive function. Individuals with higher BMI tend to have lower scores on executive function tests and thinner cerebral cortices, while individuals with thinner cerebral cortex tend to have lower scores on executive function tests. The association between BMI and executive function may be mediated by their shared relationship with the thickness of a subset of regions in PFC.

### Demographic Variables, BMI, and Executive Function

As expected from prior literature, many demographic and biological variables were related to BMI and executive function, supporting their inclusion as covariates in our statistical model.

There was no association between BMI*_Z_* and age; however, males were significantly heavier than females (β = 0.1, *t* = 2.4, *P* = 0.02) (see Table 1). Birth weight was significantly associated with BMI*_Z_* (β =0.1, *t* = 5.9, *P* < 0.001), as was household income (*F*_(2, 2387)_ = 52, *P* < 0.001), race (*F*_(3, 3286)_ = 49, *P* < 0.001), and level of parental education (*F*_(2, 2387)_ = 21, *P* < 0.001) (see Table 1). In line with previous analysis, in-scanner motion was positively associated with BMI*_Z_* (β = 0.16, *t* = 7.6, *P* < 0.001), while self-reported levels of physical activity were negatively associated with increasing BMI*_z_* (β = −0.07, *t* = 3, *P* = 0.001). There was no association between BMI*_Z_* and total brain volume; however, BMI*_Z_* was positively associated with salivary DHEA levels (β =0.17, *t* = 6, *P* < 0.001), suggesting that increased BMI was associated with more advanced pubertal stages.

**Table 1:** Demographics and variables by BMI class

	Underweight (<5th)	Lean (5th—85th)	Overweight (85th—95th)	Obese (>95th)
*N*	127	2197	472	501
Age (months)	122	120	120	120
Sex (F/M)	77/50	1113/1084	225/247	231/270
Birth weight (lbs)	6 (na = 3)	6.5 (na = 68)	6.7 (na = 18)	6.7 (na = 23)
Income (lower/middle/higher)	6/49/64 (na = 8)	250/748/1045 (na = 154)	98/167/162 (na = 45)	123/209/133 (na = 36)
Race (white/black/Hispanic/other)	92/7/14/14	1501/199/334/163	236/74/135/27	218/113/141/29
FWD	0.19 (na = 4)	0.24 (na = 164)	0.28 (na = 54)	0.31 (na = 51)
Parental education (high school/college/postgrad.)	10/75/42	215/1289/693	87/283/102	102/311/88
DHEA (pg/mL)	64 (na = 57)	61 (na = 1044)	65 (na = 216)	84 (na = 231)
Physical activity (no. days)	3.7	3.8 (na = 1)	3.5	3.5 (na = 2)
Executive function (age-corrected)	99.6 (na = 12)	99.7 (236)	95.8 (na = 52)	93.9 (na = 57)
Brain volume (cm^3^)	1180 (na = 30)	1224 (na = 550)	1216 (na = 131)	1207 (na = 112)

BMI was classified using percentile growth charts stratified according to age based on CDC 2001 look up tables (CDC Growth Charts 2018, https://www.cdc.gov/growthcharts/html_charts/bmiagerev.htm). For statistical assessment, household income levels were categorized as less than $35 000, less than $100 000, and greater than $100 000. Race was categorized as white, black, Hispanic and other. Parental education was categorized as up to and included General education diploma (GED), up to and including college or associated degrees, and postgraduate. Mean FWD was used as a measure of head motion during scanning. Physical activity was a self-reported record of number of days in past week where the subject was physically active for more than 60 min/day.

Executive abilities were significantly associated with age (β = 0.09, *t* = 4.5, *P* < 0.001) and slightly higher in females (β = 0.1, *t* = 2.4, *P* = 0.02). Birth weight was not associated with executive abilities; however, total brain volume (β = 0.07, *t* = 3.3, *P* < 0.001), in-scanner motion (β = 0.2, *t* = 7.5, *P* < 0.001), physical activity (β = 0.05, *t* = 2.2, *P* = 0.03), parental education (*F*_(2, 2387)_ = 37, *P* < 0.001), household income (*F*_(2, 2387)_ = 46, *P* < 0.001), and race (*F*_(2, 2386)_ = 27, *P* < 0.001) were associated. Overall, there was also a positive association between levels of DHEA and executive abilities (β = 0.09, *t* = 2.9, *P* = 0.005).

### Relationship Between BMI and Executive Function

**Figure 1:**
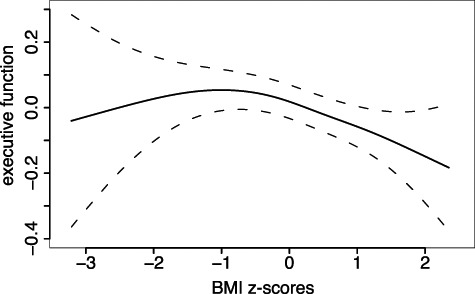
Relationship between BMI and executive function after adjustment for age, sex, race, birth weight, in-scanner motion, parental education, and household income.

There was a significant negative relationship between BMI*_Z_* and age-corrected executive function (β = −0.05, *t* = 2.4, *P* = 0.02, *n* = 2389) accounting for other variables except cortical thickness and levels of pubertal hormones (see Fig. 1). When levels of DHEA were taken into account, the relationship between BMI and executive function was reduced to trend-level (β = −0.05, *t* = 1.6, *P* = 0.1). Because there were far fewer subjects with levels of DHEA (*n* = 1227), and the effect size is identical, this likely reflects reduced power in the smaller dataset rather than DHEA mediating the relationship between BMI and executive function. Both waist circumference and waist-to-height ratio were also negatively associated with executive function (see Supplementary Materials).

### Relationship Between BMI and Cortical Thickness

Across individuals with complete data (*n* = 2668), there was a significant, negative association between BMI*_Z_* and mean global cortical thickness (β = −0.5, *t* = 2.6, *P* = 0.01) accounting for demographic and other covariates excepting DHEA.

**Figure 2:**
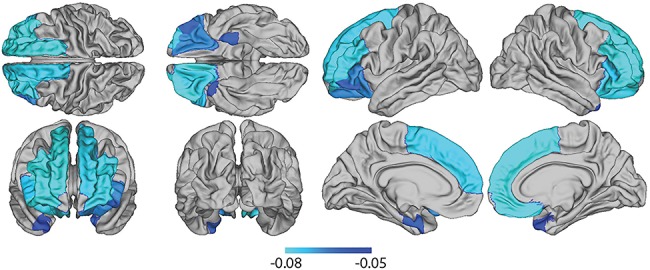
Map of reduced cortical thickness (beta regression coefficients) associated with BMI, adjusted for demographic and other confounder variables.

After FDR correction for multiple comparisons, regions of significant cortical thickness reductions bilaterally included lateral OFC, inferior frontal gyrus (parsorbitalis and pars triangularis), and rostral middle frontal and superior frontal cortex. In the left hemisphere, additional significant differences were found in entorhinal cortex, while in the right hemisphere additional changes were found in medial OFC and temporal pole (see Fig. 2, Table 1, Supplementary Material). In all cases, changes took the form of a decrease in cortical thickness associated with BMI*_Z_*.

When we repeated our analysis using waist circumference and waist-to-height ratio in place of BMI, we found that the relationship to cortical thickness was broadly similar to the pattern seen with BMI (see Supplementary Table 1 and Supplementary Materials).

In a separate analysis, there was a negative association between DHEA level and average global cortical thickness (β = −0.06, *t* = 2.2, *P* = 0.03) in line with the hypothesis that increases in pubertal hormonal levels are associated with maturation-related cortical thinning in this age range. In a regional analysis of the association between cortical thickness and BMI taking DHEA levels into account, only cortical thickness in rostral middle frontal cortex was associated with BMI (β = −0.13, *t* = 3.8, *P* = 0.01). Given that the effect size was not decreased compared with the analysis in the larger dataset, this suggests that DHEA was not a significant confound of the relationship between BMI and cortical thickness, and that differences in results were instead more likely due to a comparative lack of power in the smaller dataset (see Supplementary Table 2 and Supplementary Material).

### Relationship Between Cortical Thicknessand Executive Function

**Figure 3:**
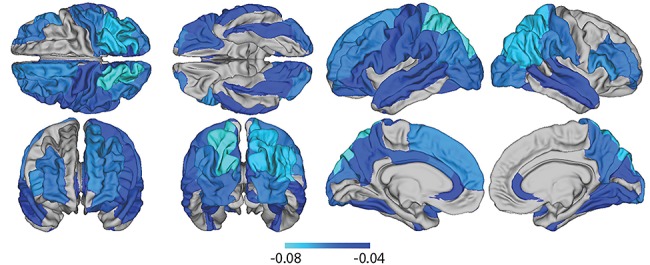
Map of reduced cortical thickness (beta regression coefficients) associated with executive function adjusted for demographic and other confounder variables.

Across individuals with complete data (*n* = 2389), there was a significant negative relationship between mean global cortical thickness and executive abilities (β = −0.07, *t* = 3.1, *P* = 0.002), without adjusting for BMI*_Z_*. At a local level and after FDR-correction, mean cortical thickness in several regions was predictive of executive function (see Fig. 3), including cuneus, fusiform, lateral occipital, rostral anterior cingulate, rostral middle frontal gyrus, superior and inferior parietal cortex, middle and superior temporal gyrus, pars opercularis, pars triangularis, postcentral, and supramarginal cortices bilaterally, and additionally the caudal anterior cingulate, superior temporal sulcus, caudal middle frontal gyrus, lateral orbitofrontal cortex, pars orbitalis, precuneus, precentral sulcus, posterior cingulate and the superior frontal cortex in the left hemisphere, and lingual region, the precuneus, and the transverse temporal sulcus in the right hemisphere (see Supplementary Table 1 and Supplementary Material). In all regions, this association took the form of a negative relationship between cortical thickness and executive function. This is in line with previous results for this age-range (Shaw et al. 2006).

Taking DHEA levels into account in the subset of the sample, the negative relationship between global mean cortical thickness and executive abilities remained (β = −0.08, *t* = 2.6, *P* = 0.02). At a regional level, executive function was again associated with reduced cortical thickness in several regions including cuneus and superior parietal cortex in the left hemisphere and pars triangularis and transverse temporal cortex in the right hemisphere. Again, the effect size was not reduced compared to the larger dataset suggesting that DHEA was not a significant confound of the relationship between cortical thickness and executive function (see Supplementary Table 2 and Supplementary Material).

### Mediation

Having established a relationship between (1) BMI and cortical thickness, (2) cortical thickness and executive function, and (3) BMI and executive function, we next examined whether cortical thickness was a significant mediator of the relationship between BMI*_Z_* and executive function.

**Figure 4:**
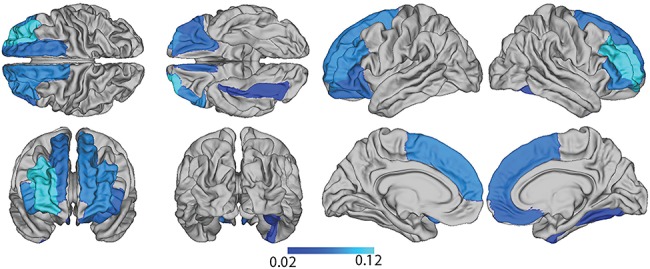
Estimate of mediation effect of regional cortical thickness on the relationship between BMI and executive function.

Results of analysis revealed that while global mean cortical thickness was not a significant mediator between BMI and executive function, cortical thickness in 11 regions partially mediates the relationship (see Fig. 4, Supplementary Table 3 and Supplementary Material). These regions included the parsorbitalis, pars triangularis, rostral middle frontal and superior frontal cortex bilaterally, and additionally lateral OFC in the left hemisphere, and fusiform and medial OFC in the right hemisphere (see Supplementary Table 3 and Supplementary Material).

## Discussion

In this study, we investigated the association between BMI, cortical thickness, and executive function in 2700 9–11 year olds recruited as part of the ABCD NIH study. We observed a negative association between executive function and cortical thickness across the cortical surface. Increased BMI was associated with lower scores on a composite measure of executive function. We also found significant BMI-related differences in cortical thickness in line with similar studies (Maayan et al. 2011; Yau et al. 2014; Ross et al. 2015). In particular, reduced cortical thickness was pronounced in orbitofrontal cortex, ventromedial PFC, and DPFC, regions involved in executive functions including decision-making, response inhibition, working memory, and cognitive flexibility.

The changes that such reduced cortical thickness reflects are unknown. For example, previous studies have suggested that MR-based measures of changes in cortical thickness during childhood may reflect, in part, increases in cortical myelination, particularly in frontal association areas (Croteau-Chonka et al. 2016). Interpreting the results of the current study along these lines, reduced cortical thickness associated with childhood obesity may be a function of an increase in cortical myelination. Future studies may consider more direct measures of myelination with a view to increasing power to detect the extent of structural mediation between BMI and executive function. Moreover, other brain parameters may also be important. For example, relative change in degree of connectivity or consolidation of activity may more closely index the development of executive function and thus may be more sensitive to BMI-related differences (Durston et al. 2006; Ezekiel et al. 2013).

Previous studies have reported a degree of regional-specificity to changes in cortical structure in relation to childhood obesity. The current study, capitalizing on a uniquely large dataset, demonstrates that increased BMI is associated with pervasive reductions in cortical thickness across much of the PFC. While our study does not allow a clear mechanistic interpretation of this predominance of effect in PFC, one possibility is that, since this region is associated with top-down control and inhibitory processes, then BMI-related changes could in turn lead to further difficulties in resisting external drives to consumption and attenuated learning from experience. This could entail a positive feedback in which early detrimental changes to PFC structure and function lead to ensuing behavioral changes that exacerbate weight gain.

More generally, PFC is involved in top-down regulation and inhibitory control as well as emotion and motivation regulation, and changes in this area are convincingly related to risk-taking behavior and substance abuse (Goldstein and Volkow 2011). In addition, the relatively extended maturational trajectory of PFC is thought to subserve experience-dependent learning (Romine and Reynolds 2005). As such, differences in PFC structure during early adolescence may possibly increase the vulnerability of this region to external stressors. Thus, BMI-associated brain changes in PFC may be regarded as a risk factor to the developing brain.

In a complementary analysis, we found that reduced cortical thickness in the PFC partially mediated the relationship between BMI and executive function. This observation is compatible with the idea that elevated BMI causes cortical thinning in turn leading to a reduction in executive function score. The direction of this causality model is supported by some observational studies. For example, in adult populations, a significant number of studies have suggested that obesity may play a causal role in the onset of brain structural changes and cognitive decline (Bruce-Keller et al. 2009; Arnoldussen et al. 2014). It is hypothesized that factors related to increased body mass such as an elevated inflammatory response or neuroendocrine dysfunction might impact on brain structure and cognitive function in a manner akin to neurodegenerative processes observed with aging. Indeed, many studies have associated increased BMI in midlife with increased rates of neurodegeneration and a significant elevated risk of dementia and Alzheimer’s disease in old age (Singh-Manoux et al. 2017). In children, a large-scale longitudinal analysis of early childhood development reported that obesity in very early childhood is a risk factor for reduced cognitive function years later (Li et al. 2018). Important corollaries to these studies are reports of significant improvement of memory and executive function following weight-loss (Gunstad et al. 2011; Veronese et al. 2016), as well as the general neuroprotective effects of severe caloric restriction (Colman et al. 2009).

However, care must be taken when interpreting our results, and we note that the causal model of BMI impacting cortical thickness which then further impacts executive function is just one of a possible six models and it is not possible to distinguish these statistically. For example, our data may also fit a model whereby BMI impacts executive function, which in turn impacts cortical thickness. Alternatively, our data would be equally compatible with a hypothesis that executive function influences BMI, which in turn may influence cortical structure. Indeed, various studies support such a hypothesis. For example, longitudinal studies suggest that the early cognitive environment may be a risk factor for developing obesity in later childhood, with children in lower cognitive stimulation environments at a 2-fold greater risk of developing obesity (Strauss and Knight 1999). Meanwhile, in bariatric patients, executive function has been shown to predict postsurgical weight-loss (Spitznagel et al. 2013).

We also acknowledge the possibility that there is no causal relationship between BMI and executive function. This would be compatible with a model in which cortical structural features drive altered BMI and executive functioning independently. This is feasible given that the genes associated with obesity-risk are predominantly and significantly expressed in the central nervous system and linked to basic functions such as glutamate signaling and synaptic function (Locke et al. 2015) and that BMI, brain structure, and various aspects of cognitive function share common genetic influences (Curran et al. 2013; Hagenaars et al. 2016; Marioni et al. 2016). The finding that BMI shares common genetic influences with various aspects of brain structure and cognition highlights the difficulty in isolating causal associations in noninterventional studies and underscores the importance of more direct studies in nonhumans. In this regard, it may be that BMI and executive function are not causally related, and structural changes associated with each may simply be regarded as an important confound of the relationship (MacKinnon et al. 2000). Indeed, we note that it is not possible to statistically distinguish between a confound and a mediator. In this regard, the results of this study suggest that BMI, cortical thickness or executive function should be included as a potential confound in any future analysis that seeks to investigate the relationship between the other 2 variables.

There were a number of significant limitations in this study. Primarily, due to the cross-sectional nature of our data, we were not able to distinguish between different possible models to determine the causal relationship between BMI, executive function, and cortical thickness. This may be addressed by future studies based on longitudinal data. In addition, our analysis was based on measures of BMI. While BMI is the most commonly used index of adiposity, it is less directly related to cardio-metabolic risks than other metrics such as waist circumference and waist-to-height ratio (Sharma et al. 2015). When we repeated our analysis for waist circumference and waist-to-height ratio, we found that both measures were associated with lower levels of executive function, as well as regional reductions in cortical thickness in a manner similar to what we observed using BMI. However, both measures additionally identified regions where increased waist circumference and waist-to-height ratio were associated with increases in cortical thickness. These results illustrate that BMI may not capture the total variation of cortical structure with increased adiposity. Finally, although we have confined this extensive analysis to the cortical sheet, we acknowledge that subcortical structures have also been implicated in obesity. Therefore, it will be important for future work to extend such analyses to subcortical regions and, critically, to examine covariance relationships between key cortical and subcortical structures in order to more fully characterize the relationship between childhood obesity and brain structure.

## Conclusions

In a large, population-based cohort, reduction in PFC cortical thickness was associated with childhood obesity. Higher BMI was also associated with reduced scores on a composite cognitive measure reflecting executive processes, and a complementary mediation analysis was consistent with cortical thickness change mediating the relationship between BMI and executive functioning. The data are consistent with a mechanism whereby PFC changes in childhood obesity may lead to altered regulation of inhibitory control and risk-taking behavior and further difficulties in weight control. However, due to the limitations of our data, care must be taken in interpreting our results and follow-up studies will be critical to establishing causal pathways between BMI, brain structure, and executive function, as well as determining if longitudinal changes in BMI have a measurable impact on these traits.

## Funding

The Bernard Wolfe Health Neuroscience Fund and the Wellcome Trust (grant number RNAG/259).

## Notes

Data used in the preparation of this article were obtained from the Adolescent Brain Cognitive Development (ABCD) Study (https://abcdstudy.org), held in the NIMH Data Archive (NDA). This is a multisite, longitudinal study designed to recruit more than 10,000 children aged 9-10 and follow them over 10 years into early adulthood. The ABCD Study is supported by the National Institutes of Health and additional federal partners “under award numbers U01DA041022, U01DA041028, U01DA041048, U01DA041089, U01DA041106, U01DA041117, U01DA041120, U01DA041134, U01DA041148, U01DA041156, U01DA041174, U24DA041123, and U24DA041147”. A full list of supporters is available at https://abcdstudy.org/nih-collaborators. A listing of participating sites and a complete listing of the study investigators can be found at https://abcdstudy.org/principal-investigators.html. ABCD consortium investigators designed and implemented the study and/or provided data but did not necessarily participate in analysis or writing of this report. This manuscript reflects the views of the authors and may not reflect the opinions or views of the NIH or ABCD consortium investigators.

The ABCD data repository grows and changes over time. The ABCD data used in this report came from the curated annual release 1.0 (DOI 10.15154/1412097). DOIs can be found at https://ndar.nih.gov/study.html?id=500.

*Conflict of Interest:* P.C.F. has received money in the past for ad hoc consultancy services to GlaxoSmithKline. All other authors declare no competing financial interests.

## Supplementary Material

Supplementary_Material_Accepted_bhz257
