# Supplementary material for: Childhood Obesity, Cortical Structure, and Executive Function in Healthy Children
Source: Cereb Cortex. 2019 Oct 24;30(4):2519–28. doi: 10.1093/cercor/bhz257 (PMC7175011; doi:10.1093/cercor/bhz257)
Supplement: Supplementary_Material_Accepted_bhz257 [file supplementary_material_accepted_bhz257.docx]

**Supplementary Material**

**Waist-circumference, waist-to-height ratio and executive function:**

Full data on waist circumference and waist-to-height ratio were available for n = 2667 subjects.

Waist circumference was not significantly related to average global cortical thickness, birth weight, parental education or levels of household income, but it was related to age (β =-0.11, t = 5.5, p < 0.001), sex (β =-0.29, t = 6.7, p < 0.0001), motion (β =-0.05, t = 2.3, p = 0.02), total brain volume (β =0.16, t = 7.3, p < 0.0001), and race (F_(3, 2677)_= 25, p < 0.001).

Increased waist circumference was associated with lower executive function at a trend-level (β =-0.03, t = 1.6, p = 0.1) based on data for 2393 subjects.

WHR was not significantly related to average global cortical thickness, parental education or household income, but was related to age (β =-0.12, t = 6.1, p < 0.001), sex (β =-0.23, t = 5.4, p < 0.0001), motion (β =-0.13, t = 6.7, p < 0.0001), total brain volume (β =0.15, t = 6.7, p < 0.0001), and race (F_(3, 2677)_= 46, p < 0.001).

Increased waist-to-height ratio was associated with lower executive function (β =-0.06, t = 2.8, p = 0.006), based on data from 2387 subjects.

**Supplementary Table S1**: Regression coefficients for various regression models, namely executive function (EF) regressed on cortical thickness (CT); cortical thickness regressed on z-scores of BMI, waist-to-height ratio (WHR), waist-circumference (WC) respectively. Z-scores for each parameter were based on published data (Sharma et al., 2015). P-values for regression were adjusted for multiple comparisons using FDR.

|  | **EF ~ CT** | | **CT ~ BMI** | | **CT ~ WHR** | | **CT ~ WC** | |
| --- | --- | --- | --- | --- | --- | --- | --- | --- |
| **Region** | β **(CT)** | **P_adj_ (CT)** | β **(BMI)** | **P_adj_ (BMI)** | β **(WHR)** | **P_adj_ (WHR)** | β **(WC)** | **P_adj_ (WC)** |
| CT_LH_BANKSSTS | -0.051 | 0.03 |  |  |  |  |  |  |
| CT_LH_CAUDALANTERIORCING | -0.058 | 0.02 |  |  | 0.081 | 0.001 | 0.063 | 0.012 |
| CT_LH_CAUDALMIDDLEFRONT | -0.06 | 0.02 |  |  |  |  |  |  |
| CT_LH_CUNEUS | -0.057 | 0.023 |  |  |  |  |  |  |
| CT_LH_ENTORHINAL |  |  | -0.059 | 0.018 |  |  | -0.052 | 0.044 |
| CT_LH_FUSIFORM | -0.048 | 0.04 |  |  |  |  |  |  |
| CT_LH_INFERIORPARIET | -0.06 | 0.02 |  |  |  |  |  |  |
| CT_LH_LTR_OCCIPITAL | -0.056 | 0.03 |  |  | 0.078 | 0.001 | 0.06 | 0.012 |
| CT_LH_LTR_ORBITOFRONT | -0.052 | 0.03 | -0.066 | 0.006 |  |  | -0.063 | 0.012 |
| CT_LH_MEDIALORBITOFRONTAL |  |  |  |  |  |  | -0.079 | 0.001 |
| CT_LH_MIDDLETEMP | -0.048 | 0.036 |  |  |  |  |  |  |
| CT_LH_PARSOPERCULARIS | -0.052 | 0.03 |  |  |  |  |  |  |
| CT_LH_PARSORBITALIS | -0.048 | 0.035 | -0.059 | 0.018 |  |  |  |  |
| CT_LH_PARSTRIANGULARIS | -0.05 | 0.035 | -0.066 | 0.007 |  |  |  |  |
| CT_LH_POSTCENTRAL | -0.053 | 0.03 |  |  | 0.057 | 0.029 |  |  |
| CT_LH_POSTERIORCING | -0.052 | 0.03 |  |  |  |  |  |  |
| CT_LH_PRECENTRAL | -0.048 | 0.039 |  |  |  |  |  |  |
| CT_LH_PRECUNEUS | -0.05 | 0.035 |  |  |  |  |  |  |
| CT_LH_ROSTRALANTERIORCING | -0.045 | 0.049 |  |  |  |  |  |  |
| CT_LH_ROSTRALMIDDLEFRONT | -0.06 | 0.02 | -0.078 | 0.001 |  |  |  |  |
| CT_LH_SUPERIORFRONT | -0.063 | 0.02 | -0.076 | 0.001 |  |  |  |  |
| CT_LH_SUPERIORPARIET | -0.08 | 0.006 |  |  |  |  |  |  |
| CT_LH_SUPERIORTEMP | -0.05 | 0.035 |  |  |  |  |  |  |
| CT_LH_SUPRAMARGINAL | -0.051 | 0.03 |  |  |  |  |  |  |
| CT_LH_TEMPORAL |  |  |  |  |  |  | -0.061 | -0.016 |
| CT_RH_CUNEUS | -0.048 | 0.042 |  |  |  |  |  |  |
| CT_RH_FUSIFORM | -0.047 | 0.044 |  |  |  |  |  |  |
| CT_RH_INFERIORPARIET | -0.072 | 0.009 |  |  |  |  |  |  |
| CT_RH_LTR_OCCIPITAL | -0.06 | 0.023 |  |  | 0.073 | 0.002 | 0.053 | 0.031 |
| CT_RH_LTR_ORBITOFRONT |  |  | -0.077 | 0.001 | -0.058 | 0.029 | -0.078 | 0.001 |
| CT_RH_LINGUAL | -0.05 | 0.037 |  |  |  |  |  |  |
| CT_RH_MEDIALORBITOFRONT |  |  | -0.086 | 0 | -0.071 | 0.006 | -0.095 | 0 |
| CT_RH_MIDDLETEMP | -0.046 | 0.044 |  |  |  |  |  |  |
| CT_RH_PARSOPERCULARIS | -0.062 | 0.02 |  |  |  |  |  |  |
| CT_RH_PARSORBITALIS |  |  | -0.087 | 0 | -0.063 | 0.022 | -0.075 | 0.003 |
| CT_RH_PARSTRIANGULARIS | -0.063 | 0.02 | -0.073 | 0.002 |  |  |  |  |
| CT_RH_POSTCENTRAL | -0.058 | 0.022 |  |  |  |  |  |  |
| CT_RH_PRECUNEUS | -0.057 | 0.023 |  |  |  |  |  |  |
| CT_RH_ROSTRALANTERIORCING | -0.044 | 0.049 |  |  |  |  |  |  |
| CT_RH_ROSTRALMIDDLEFRONT | -0.058 | 0.02 | -0.125 | 0 |  |  | -0.067 | 0.01 |
| CT_RH_SUPERIORFRONT |  |  | -0.092 | 0 |  |  | -0.055 | -0.031 |
| CT_RH_SUPERIORPARIET | -0.072 | 0.009 |  |  |  |  |  |  |
| CT_RH_SUPERIORTEMP | -0.05 | 0.035 |  |  |  |  |  |  |
| CT_RH_SUPRAMARGINAL | -0.06 | 0.02 |  |  | 0.06 | 0.026 | 0.055 | 0.031 |
| CT_RH_TEMPPOLE |  |  | -0.055 | 0.028 |  |  |  |  |
| CT_RH_TRANSVERSETEMP | -0.045 | 0.05 |  |  |  |  |  |  |

**Supplementary Table 2**: Regression coefficients for various regression models including DHEA as a covariate, namely executive function (EF) regressed on cortical thickness (CT); cortical thickness regressed on BMIz-scores. P-values were adjusted for multiple comparisons using FDR. * indicates statistical significance at α = 0.05, all other results for trend level α < 0.1

|  | **EF ~ CT** | | **CT ~ BMI** | |
| --- | --- | --- | --- | --- |
|  | β **(CT)** | **P_adj_ (BMI)** | β **(CT)** | **P_adj_ (BMI)** |
| CT_LH_BANKSSTS | -0.072 | 0.053 |  |  |
| CT_LH_CAUDALMIDDLEFRONT | -0.058 | 0.085 |  |  |
| CT_LH_CUNEUS | -0.091 | 0.026* |  |  |
| CT_LH_ENTORHINAL |  |  | -0.08 | 0.053 |
| CT_LH_INFERIORPARIET | -0.073 | 0.053 |  |  |
| CT_LH_LTR_OCCIPITAL | -0.083 | 0.053 |  |  |
| CT_LH_LTR_ORBITOFRONT | -0.078 | 0.053 |  |  |
| CT_LH_MEDIALORBITOFRONTAL |  |  |  |  |
| CT_LH_MIDDLETEMP | -0.06 | 0.085 |  |  |
| CT_LH_PARSOPERCULARIS | -0.073 | 0.053 |  |  |
| CT_LH_PARSORBITALIS | -0.058 | 0.085 |  |  |
| CT_LH_PARSTRIANGULARIS | -0.058 | 0.085 | -0.073 | 0.076 |
| CT_LH_PRECUNEUS | -0.066 | 0.079 |  |  |
| CT_LH_ROSTRALMIDDLEFRONT | -0.068 | 0.074 |  |  |
| CT_LH_SUPERIORFRONT | -0.06 | 0.085 |  |  |
| CT_LH_SUPERIORPARIET | -0.091 | 0.026* |  |  |
| CT_LH_SUPERIORTEMP | -0.061 | 0.085 |  |  |
| CT_RH_CUNEUS | -0.06 | 0.085 |  |  |
| CT_RH_INFERIORPARIET | -0.074 | 0.053 |  |  |
| CT_RH_LTR_OCCIPITAL | -0.077 | 0.053 |  |  |
| CT_RH_LINGUAL | -0.066 | 0.079 |  |  |
| CT_RH_MEDIALORBITOFRONT |  |  | -0.074 | 0.076 |
| CT_RH_PARSORBITALIS |  |  | -0.08 | 0.053 |
| CT_RH_PARSTRIANGULARIS | -0.104 | 0.014* |  |  |
| CT_RH_PRECUNEUS | -0.066 | 0.079 |  |  |
| CT_RH_ROSTRALMIDDLEFRONT | -0.062 | 0.085 | -0.105 | 0.01* |
| CT_RH_SUPERIORFRONT |  |  | -0.082 | 0.053 |
| CT_RH_SUPERIORPARIET | -0.08 | 0.053 |  |  |
| CT_RH_SUPERIORTEMP | -0.068 | 0.074 |  |  |
| CT_RH_SUPRAMARGINAL | -0.064 | 0.079 |  |  |
| CT_RH_TRANSVERSETEMP | -0.091 | 0.026* |  |  |

**Mediation**

Mediation effects are determined by first estimating the total effect of the model, which is defined as the regression of the dependent variable (e.g. executive function, EF) on the independent variable (e.g. body mass index, BMI). In the next step (the direct model), the dependent variable (EF) is regressed on the independent variable (BMI) and the mediator (cortical thickness, CT). The magnitude of the mediation effect is determined by subtracting the beta coefficients of the independent variable in the total effect model from the direct model (e.g. **β_1_ – β_4_** see Figure S1). Bootstrapping methods can be used to determine whether this change is significantly different form zero.

Figure S1. Schematic of cortical thickness as a mediator of the relationship between BMI and executive function. The magnitude of the mediation is assessed by subtracting the regression coefficients **β_1_ – β_4._**

**Supplementary Table 3**: Regional results of mediation analysis for the following models (a) CT as mediator of the relationship between BMI (independent variable) and EF (dependent variable); (b) CT as mediator of the relationship between waist circumference (WC) (independent variable) and EF (dependent variable), (c) CT as mediator of the relationship between waist-to-height ratio (WHR) (independent variable) and EF (dependent variable). P-values for mediation were generated with bootstrapping methods.

|  | **BMI - CT - EF** | | |
| --- | --- | --- | --- |
|  | Total effect (Y ~ X)  Estimate **β** (p-value) | Direct effect (Y ~ X + M) Estimate **β** (p-value) | Mediation effect  Estimate **Δβ** (p-value) |
| LH_LTR_ORBITOFRONTAL | -0.77 (0.02) | -0.84 (0.01) | 0.06 (< 0.001) |
| LH_PARSORBITALIS | -0.76 (0.02) | -0.82 (0.01) | 0.06 (0.01) |
| LH_PARSTRIANGULARIS | -0.78 (0.01) | -0.84 (< 0.001) | 0.06 (< 0.001) |
| LH_ROSTRALMIDDLEFRONTAL | -0.75 (0.01) | -0.83 (< 0.001) | 0.08 (< 0.001) |
| LH_SUPERIORFRONTAL | -0.75 (0.02) | -0.82 (0.01) | 0.07 (< 0.001) |
| RH_FUSIFORM | -0.73 (0.02) | -0.77 (0.01) | 0.04 (0.04) |
| RH_MEDIALORBITOFRONTAL | -0.75 (0.02) | -0.81 (0.01) | 0.06 (0.03) |
| RH_PARSORBITALIS | -0.77 (0.01) | -0.85 (0.01) | 0.07 (0.02) |
| RH_PARSTRIANGULARIS | -0.8 (0.01) | -0.87 (0.01) | 0.08 (< 0.001) |
| RH_ROSTRALMIDDLEFRONTAL | -0.78 (0.01) | -0.89 (< 0.001) | 0.11 (< 0.001) |
| RH_SUPERIORFRONTAL | -0.79 (0.01) | -0.85 (0.01) | 0.07 (0.02) |
|  | **WC - CT - EF** | | |
| LH_CAUDALANTERIORCINGULATE | -0.55 (0.09) | -0.5 (0.13) | -0.05 (0.02) |
| LH_LTR_OCCIPITAL | -0.55 (0.09) | -0.49 (0.13) | -0.05 (0.01) |
| LH_POSTCENTRAL | -0.52 (0.11) | -0.48 (0.13) | -0.04 (0.05) |
| RH_LTR_OCCIPITAL | -0.55 (0.09) | -0.5 (0.12) | -0.05 (0.01) |
| RH_MEDIALORBITOFRONTAL | -0.54 (0.09) | -0.61 (0.07) | 0.07 (0.03) |
| RH_PARSORBITALIS | -0.54 (0.07) | -0.62 (0.04) | 0.08 (0.01) |
| RH_SUPRAMARGINAL | -0.52 (0.11) | -0.47 (0.13) | -0.05 (0.02) |
|  | **WHR - CT - EF** | | |
| LH_CAUDALANTERIORCINGULATE | -0.93 (0.01) | -0.863 (0.01) | -0.06 (0.01) |
| LH_LTR_OCCIPITAL | -0.93 (0.01) | -0.87 (0.02) | -0.07 (< 0.001) |
| LH_LTR_ORBITOFRONTAL | -0.093 (< 0.001) | -0.98 (< 0.001) | 0.05 (0.03) |
| LH_MEDIALORBITOFRONTAL | -0.94 (< 0.001) | -0.99 (< 0.001) | 0.05 (0.03) |
| RH_LTR_OCCIPITAL | -0.93 (< 0.001) | -0.87 (0.01) | -0.06 (< 0.001) |
| RH_MEDIALORBITOFRONTAL | -0.94 (< 0.001) | -1 (< 0.001) | 0.06 (0.02) |
| RH_PARSORBITALIS | -0.93 (0.01) | -1 (< 0.001) | 0.08 (0.01) |
